# STAT3 Pathways Contribute to β-HCH Interference with Anticancer Tyrosine Kinase Inhibitors

**DOI:** 10.3390/ijms25116181

**Published:** 2024-06-04

**Authors:** Sara Fiorini, Elisabetta Rubini, Monia Perugini, Fabio Altieri, Silvia Chichiarelli, Giorgia Meschiari, Giulia Arrighetti, John Vijgen, Pier Giorgio Natali, Marco Minacori, Margherita Eufemi

**Affiliations:** 1 Department of Biochemical Science “A. Rossi Fanelli”, Faculty of Pharmacy and Medicine, Sapienza University of Rome, P.le Aldo Moro 5, 00185 Rome, Italy; sara.fiorini@uniroma1.it (S.F.); fabio.altieri@uniroma1.it (F.A.); silvia.chichiarelli@uniroma1.it (S.C.); giorgia.meschiari@uniroma1.it (G.M.); mminacori@unite.it (M.M.); margherita.eufemi@uniroma1.it (M.E.); 2 Institute of Molecular Biology and Pathology, CNR National Research Council, Via degli Apuli, 4, 00185 Rome, Italy; elisabetta.rubini@uniroma1.it; 3 Department of Bioscience and Agro-Food and Environmental Technology, University of Teramo, Campus “Aurelio Saliceti”, Via R. Balzarini 1, 64100 Teramo, Italy; mperugini@unite.it; 4 Department of Cultures, Politics and Society, University of Turin, Via Verdi, 8, 10124 Turin, Italy; giulia.arrighetti@unito.it; 5 International HCH and Pesticides Association (IHPA), Elmevej 14, 2840 Holte, Denmark; john.vijgen@ihpa.info; 6 Collegium Ramazzini, Castello di Bentivoglio, Via Saliceto, 3, 40010 Bologna, Italy; 7 Lega Italiana per la Lotta contro i Tumori (LILT), Associazione Metropolitana di Roma, Via Nomentana, 303, 00162 Rome, Italy

**Keywords:** STAT3, β-hexachlorocyclohexane, chemoresistance, environmental pollutants, signaling transduction

## Abstract

Organochlorine pesticides (OCPs) are a class of environmentally persistent and bioaccumulative pollutants. Among these, β-hexachlorocyclohexane (β-HCH) is a byproduct of lindane synthesis, one of the most worldwide widespread pesticides. β-HCH cellular mechanisms inducing chemical carcinogenesis correspond to many of those inducing chemoresistance, in particular, by the activation of signal transducer and activator of transcription 3 (STAT3) signaling pathways. For this purpose, four cell lines, representative of breast, lung, prostate, and hepatocellular cancers, were treated with β-HCH, specific tyrosine kinase inhibitors (TKIs), and a STAT3 inhibitor. All cell samples were analyzed by a viability assay, immunoblotting analysis, a wound-healing assay, and a colony formation assay. The results show that β-HCH reduces the efficacy of TKIs. The STAT3 protein, in this context, plays a central role. In fact, by inhibiting its activity, the efficacy of the anticancer drug is restored. Furthermore, this manuscript aimed to draw the attention of the scientific and socio-healthcare community to the issue of prolonged exposure to contaminants and their impact on drug efficacy.

## 1. Introduction

Cancer therapy chemoresistance, the ability of cancer cells to reduce the efficacy and potency of a chemotherapeutic drug, is the most critical cellular process impairing the successful outcomes of cancer medical therapies, thus representing the main challenge for cancer management [1]. Mechanisms of chemoresistance are classified as intrinsic (pre-existing) or extrinsic (acquired) [2] and may be drug-specific or involve multiple therapeutics, resulting in multi-drug resistance [3]. Intrinsic resistance can be defined as the pre-existence of resistance mechanisms before starting therapy. The reasons for its occurrence are heterogeneous and include (1) the pre-existence of therapy-resistant cell populations; (2) the patient’s low tolerance to the therapy or the occurrence of unbearable side effects; and (3) the inability of the therapy to achieve the required pharmacokinetic profile through altered absorption, distribution, metabolism, and excretion [4]. On the contrary, extrinsic resistance occurs at a later stage of treatment, being attributed to cellular mechanisms such as (1) the overexpression of anti-apoptotic proteins or efflux pumps, (2) mutations of target proteins, and (3) the synergistic activation of signaling pathways in tumor cells. This acquired chemoresistance may result from any of the above mechanisms, individually or from their synergistic concurrent effects [5].

Environmental pollutants play an important role in the context of extrinsic chemoresistance [6]. Pollutants are compounds generated by human activities that are becoming ubiquitous in the ecosystem and are responsible for approximately 9 million deaths yearly, i.e., 16% of all deaths worldwide [7]. They can bioaccumulate in the human body, triggering multiple cellular activities responsible for the onset and progression of “non-communicable disease” [8], including cardiovascular, pulmonary, metabolic, and neurodegenerative [9] diseases and cancer [10].

A wealth of scientific data suggest that exposure to environmental pollutants, even at low concentrations, can lead to an increased risk of developing cancer and/or accelerate its progression [11]. Environmental pollutants could interfere with the cell cycle and cause uncontrolled proliferation [12], inhibit the apoptotic process [13], promote angiogenesis [14], induce the epithelial–mesenchymal transition (EMT) [15], increase the secretion of metalloproteases causing the metastasization process [16], and promote genomic instability [17] and chronic inflammation [18,19].

All these cellular processes activated by pollutants coincide with those that trigger acquired chemoresistance [19]. This suggests that pollutants might contribute to a reduced therapeutic response to anticancer drugs and disease progression. For approximately a decade, our research group has been investigating the cellular and molecular effects of β-hexachlorocyclohexane (β-HCH) [20,21,22], an organochlorinated pollutant belonging to the organochlorine pesticides (OCPs) family. β-HCH is a byproduct derived from the synthesis of lindane (γ-HCH), one of the most widespread pesticides on the planet, definitively banned by the Stockholm Convention on Persistent Organic Pollutants in 2009 [23]. Due to its physicochemical properties, β-HCH is considered the “fossil isomer” within the lindane family, as it is the most persistent and bioaccumulative, both in the environment and in humans [24]. For each ton of lindane produced, 8–12 tons of unwanted derivatives are generated, resulting in an accumulation of nearly 7.2 million tons of HCH waste isomers [25]. These byproducts are predominantly still deposited in unregulated landfills at numerous sites across the globe. Our previous studies [20,21,22] and those by Papaccio et al. [26] on the molecular and cellular mechanisms of β-HCH have described its activity as an endocrine disruptor, activator of the aryl hydrocarbon receptor, activator of signal transducer and activator of transcription 3 (STAT3) signaling pathways, modulator of energy metabolism, inducing the Warburg effect, ROS species booster, and promoter of all three stages of carcinogenesis.

Since the cellular effects triggered by β-HCH are comparable to those typical of acquired chemoresistance, it has been hypothesized that β-HCH could influence the response to anticancer tyrosine kinase inhibitors (TKIs). Therefore, the aim of the present study was to investigate the impact of β-HCH on cellular responses to anticancer drugs, employing cellular targets commonly activated in breast, prostate, lung, and hepatocarcinoma tumors. Our hypothesis was that the STAT3 protein, in addition to being the hub of cellular responses to β-HCH [20], might also be involved in the onset of β-HCH-induced chemoresistance to TKIs. The role of STAT3 in resistance to anticancer drugs is a phenomenon described in several scientific studies [27,28,29,30].

## 2. Results

To replicate the real exposure conditions, the experimental concentration of 10 μM of β-HCH was extrapolated from a biomonitoring study conducted on inhabitants of the Valle del Sacco (Italy) [31]. The human cell lines included hormone-responsive, triple-positive breast cancer (MCF-7), non-small cell lung cancer (H358), human prostate carcinoma (LNCaP), and hepatocellular carcinoma (HepG2) cell lines. Anticancer drugs such as cisplatin, doxorubicin, and paclitaxel represent chemotherapeutic agents that, despite significantly contributing to revolutionizing pharmacological therapy for tumors, exhibit poor tumor selectivity and are associated with both acute and chronic toxicity [32,33]. In recent years, progress in the molecular dissection of malignant transformation and progression has identified an increasing number of potentially druggable molecular pathways, such as those regulating cell cycle progression, the induction of apoptosis, angiogenesis, and the interaction of tumor cells with the extracellular matrix [34]. Among these new targets, enzymes endowed with tyrosine kinase activity have undergone intense investigation and clinical development in view of their high efficiency, specificity, and favorable safety profiles and can be combined with other forms of chemotherapy or radiation therapy [35]. The tyrosine kinase inhibitors (TKIs) employed in this study are all commonly used as chemotherapeutic agents that target proteins within signaling pathways activated by β-HCH, as demonstrated in our previous studies [36]. Specifically, lapatinib (0.8 μM), a HER2 inhibitor [37], was used for breast cancer (MCF7); gefitinib (15 µM), an EGFR inhibitor [38], for lung cancer (H358); dasatinib (70 nM), an Src inhibitor [39], for prostate cancer (LNCaP); and finally, AZD1480 (6 μM), a JAK2 inhibitor [40], for hepatocellular carcinoma. Our hypothesis was that the STAT3 protein, in addition to being the hub of cellular responses to β-HCH [20], might also be involved in the onset of β-HCH-induced chemoresistance to TKIs. Indeed, the involvement of STAT3 in chemoresistance processes is a phenomenon described in several scientific studies [27].

### 2.1. β-HCH Counteracts Inhibitor Kinase-Induced Cytotoxicity

**Figure 1:**
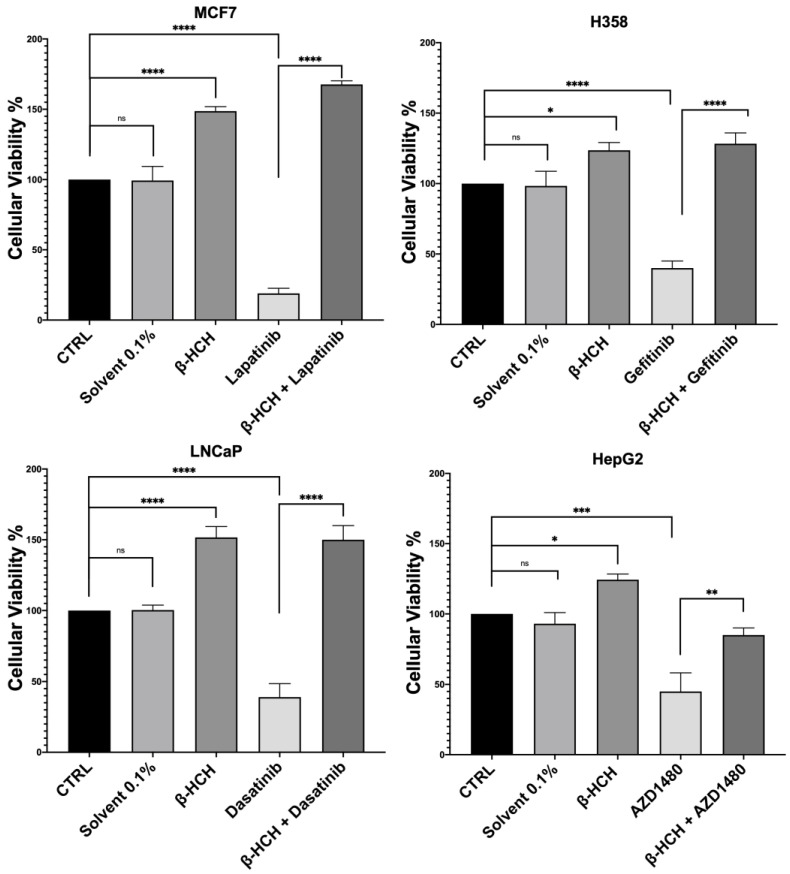
CCK-8 assay performed on MCF-7, H358, LNCaP, and HepG2. The control (CTRL) comprised untreated cells, and the solvent used was DMSO. Cells were pretreated overnight with 10 µM of β-HCH and then treated for 48 h with specific TKIs at the concentrations shown on page 3. The values reported in the histograms represent the averages of three independent experiments and are presented as the means and standard deviation. Statistical analysis was performed with GraphPad Prisma software version 8.2.1 (279) using ANOVA followed by Tukey’s post hoc test. Statistically significant differences referring to the CTRL or TKIs are marked with asterisks (ns: not statistically significant, * *p* < 0.05; ** *p* < 0.01; *** *p* < 0.001; **** *p* < 0.0001).

Firstly, to assess the influence of β-HCH on drug efficacy, a cell viability assay was conducted using CCK-8 (Figure 1).

**Figure 2:**
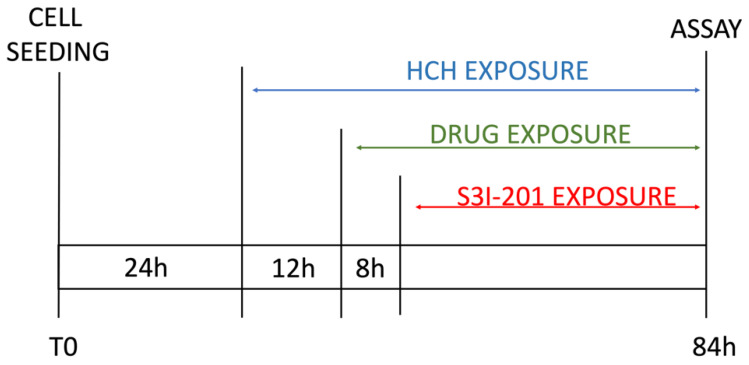
Scheme of experimental timeline.

The obtained results were similar across the four cell lines. The control (CTRL) comprised untreated cells, and the solvent used was DMSO. Samples treated with β-HCH alone confirmed our previous data [20,21,22], showing an increase in cell viability following exposure to the pollutant. However, in samples treated with the chemotherapeutic agent, there was a significant reduction in cell viability compared with the control, indicating the cytotoxicity of the anticancer drugs. Lastly, in samples pre-treated overnight with β-HCH and then incubated for 48 h with the TKIs, there was a clear increase in cell viability, especially in MCF-7, H358, and LNCaP, where the cell viability exceeded that of the control. These results provide clear evidence that pre-treatment with β-HCH counteracts the cytotoxic effect of the drugs. As stated in the preceding paragraph, the cellular effects of β-HCH are comparable to those of extrinsic chemoresistance [21]. The synergistic effect of these cellular responses induced by β-HCH, combined with its environmental persistence and bioaccumulation in the body, could contribute to it being the cause of a reduced or absent response to the TKIs, akin to a battle between David and Goliath. Furthermore, as supported by our previous study [20], the oncoprotein STAT3, which is involved in chemoresistance processes [41], plays a crucial role in the signaling pathways activated by β-HCH. To support our hypothesis that STAT3 is pivotal in reducing the effect of anticancer therapies induced by β-HCH, a viability assay in the presence of 100 μM of S3I-201, a STAT3 inhibitor [42], was performed following the experimental scheme outlined in Figure 2.

**Figure 3:**
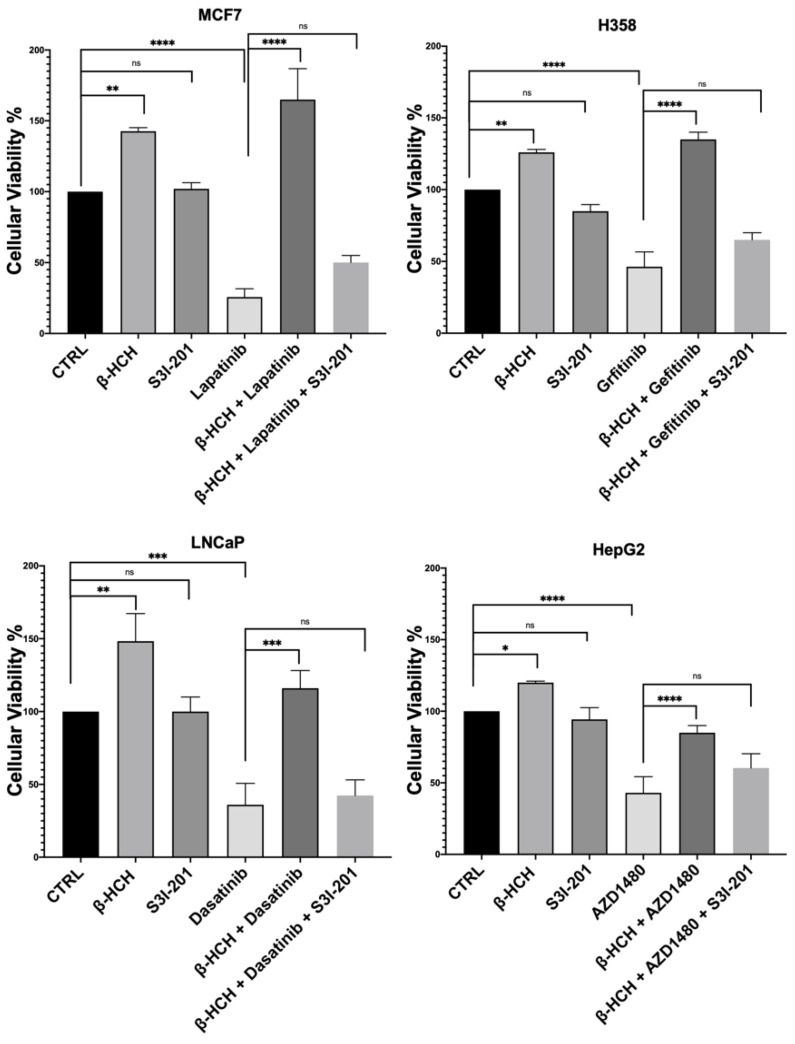
CCK-8 assay performed on MCF-7, H358, LNCaP, and HepG2. Cells were incubated with 10 µM of β-HCH, TKIs, and S3I-201, as shown in Figure 2. Cellular viability decreased after treatment with β-HCH + TKIs + S3I-201 compared with samples treated only with β-HCH + TKIs. The values reported in the histograms represent the averages of three independent experiments and are presented as the means and standard deviation. Statistical analysis was performed with the GraphPad Prisma software using ANOVA followed by Tukey’s post hoc test. Statistically significant differences referring to the CTRL or TKIs are marked with asterisks (ns: not statistically significant, * *p* < 0.05; ** *p* < 0.01; *** *p* < 0.001; **** *p* < 0.0001).

The results shown in Figure 3 confirm our hypothesis, as samples subjected to triple treatment (β-HCH + TKIs + S3I-201) exhibited a clear reduction in viability.

### 2.2. β-HCH Activates STAT3 Protein

**Figure 4:**
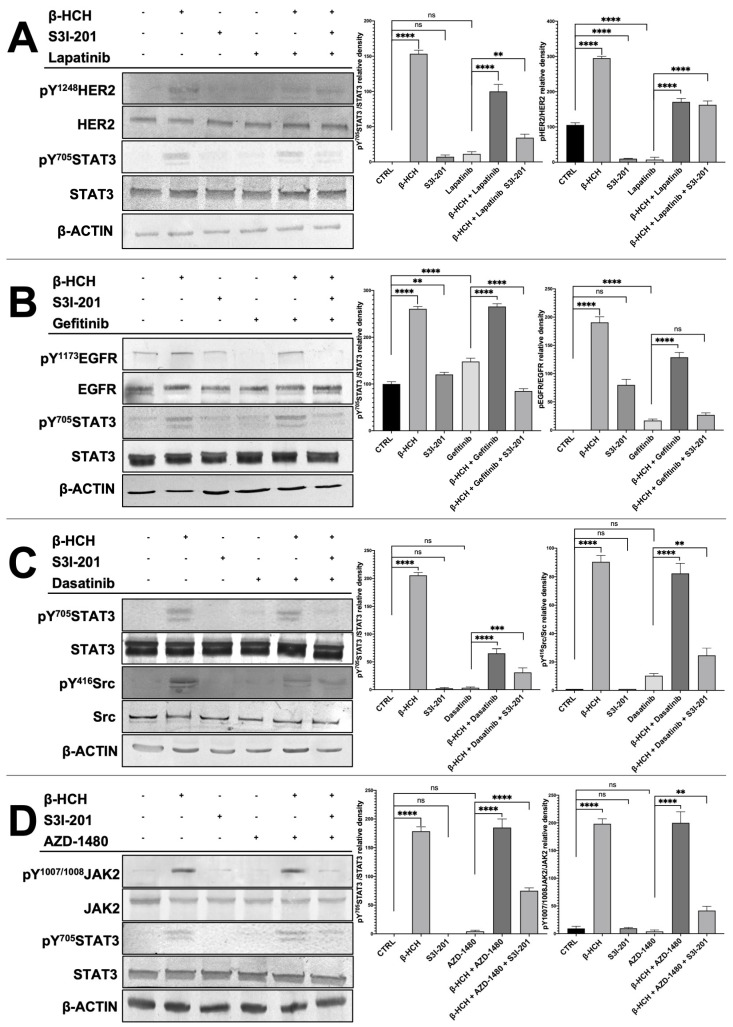
Immunoblotting evaluating the activation of STAT3 and HER2 in MCF-7 (**A**), STAT3 and EGFR in H358 (**B**), STAT3 and SRC in LNCAP (**C**), and STAT3 and JACK2 in HepG2 (**D**). Total protein extracts were subjected to immunoblot analysis. Immunoblot evidenced that STAT3 phosphorylation (pY705-STAT3), HER2 phosphorylation (pY1248-HER2), JACK2 phosphorylation (pY1007/1008-JAK2), and Src phosphorylation (pY416-Src) increased upon treatment with 10 µM of β-HCH. The increase in STAT3 phosphorylation was upheld even with the combined treatment β-HCH+ TKIs, whereas a decrease in the band intensity occurred in the sample with triple treatment (β-HCH+ TKI+ S3I-201), as evident in the relative densitometry. β-actin was used for housekeeping. Phosphorylation levels referred to the amount of total STAT3, HER2, EGFR, Src, or JAK2 present in each sample and were compared with the control. These images are representative of three independent experiments with similar results. Statistical analysis was performed with the GraphPad Prisma software using ANOVA followed by Tukey’s post hoc test. Statistically significant differences referring to the CTRL or TKIs are marked with asterisks (ns: not statistically significant, ** *p* < 0.01; *** *p* < 0.001; **** *p* < 0.0001).

To verify that the differences in cell viability were attributable to the action of the kinase inhibitors and S3I-201, total protein extracts, obtained from the differently treated four cell lines, were analyzed by Western blotting (Figure 4). For each cell line, both the STAT3 protein and the target of the pathways activated by β-HCH were investigated, analyzing phosphorylated forms, indicative of their activation, as well as non-phosphorylated forms.

The results presented in Figure 4 clearly demonstrate in all reported cellular models (MCF7, LNCaP, H358, and HepG2) the activation of the receptors HER2, SRC, EGFR, and JAK2 and the subsequent activation of the STAT3 protein via the phosphorylation of the tyrosine residue 705. On the contrary, the phosphorylation profiles of these receptors were significantly reduced in samples obtained from cells treated with only the chemotherapeutic drugs (lapatinib, gefitinib, dasatinib, and AZD1480). Differently, if the cells were pretreated with β-HCH and then with the TKIs, there was an increase in the activation of both receptors, and the STAT3 protein was present. Finally, in the triple treatment, there was a clear reduction in the activation of both receptors and the STAT3 protein.

The correlation between the Western blotting results and those obtained from the CCK-8 assay confirms that the activation of STAT3 oncogenic pathways by β-HCH contributes to the increase in cell viability and the reduction in the efficacy of TKIs in samples treated with the pollutant. Furthermore, in the triple treatment (β-HCH + TKIs + S3I-201), the high mortality associated with the almost absent activation of the STAT3 protein leads us to hypothesize that pathways mediated by the oncoprotein play a central role in reducing the efficacy of the tested drugs. To conclude, the following can be affirmed: (1) the pathways activated by β-HCH are consistent with those demonstrated in our previous studies, (2) the TKIs we used exert a specific inhibitory action against the target proteins of β-HCH, (3) β-HCH reduces the efficacy of the tested drugs, and (4) the STAT3 protein, in this context, plays a central role. In fact, by inhibiting its activity, the efficacy of the anticancer drug is restored.

### 2.3. β-HCH Counteracts TKIs’ Effects on Cell Migration

**Figure 5:**
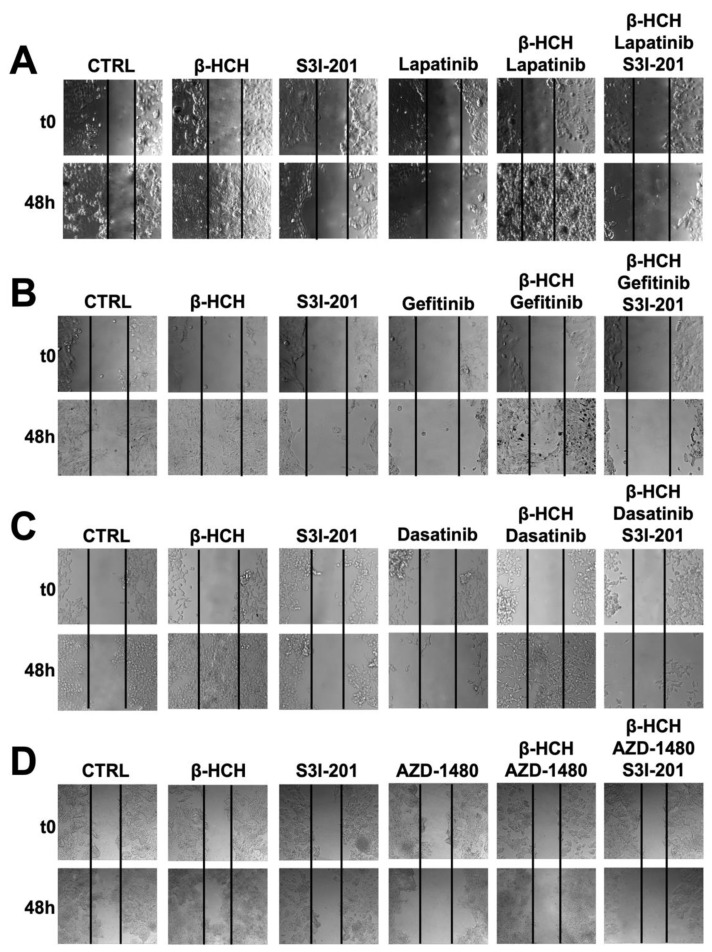
Wound-healing assay conducted on MCF-7 (**A**), H358 (**B**), LNCaP (**C**), and HePG2 (**D**) cell lines. Images were collected immediately after scratching the cell monolayer (T0) and 48 h post-treatment with TKIs. The results show that after 48 h of incubation with specific TKIs +10 µM of β-HCH, the pollutant affected the drug efficacy. Conversely, in the triple treatment (β-HCH+ TKI+ S3I-201), by inhibiting the STAT3 protein, there was a reduction in the cellular migratory capability. These images, representative of three independent experiments with similar results, were captured with a Leica AF6000 Modular System microscope.

As further confirmation of β-HCH’s ability to influence the efficacy of TKIs, a wound-healing assay was performed (Figure 5).

**Figure 6:**
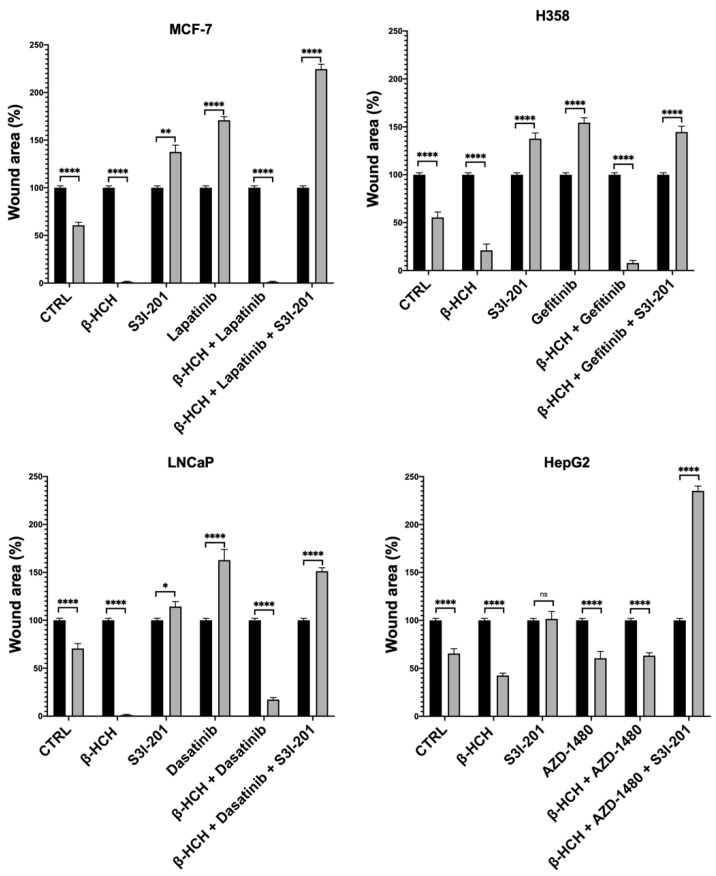
Quantification of cell migration rate at 0 h and 48 h in wound-healing assay in Figure 5. The wound area was analyzed by using ImageJ version number 1.53a. The results are reported as the means ± SE of the percentages of three independent experiments (one-way ANOVA followed by Tukey’s post hoc test: ns: not statistically significant, * *p* < 0.05; ** *p* < 0.01; **** *p* < 0.0001) vs. the wound area percentage at T0.

This technique aims to measure the migratory capacity of cells, a crucial characteristic in advanced- and undifferentiated-stage tumors [43]. Figure 5 and Figure 6 clearly show that samples treated with β-HCH alone exhibited a higher migratory capacity compared with the control. Treatment with TKIs reduced cell migration, while in treatments with S3I-201, there were no significant differences compared with the control. Cell migration further increased in the co-treatment (β-HCH + TKIs), while, in line with our hypothesis, there was a significant reduction in migratory ability in the triple treatment with the STAT3 protein inhibitor. The latest results confirm the key role of the STAT3 protein in β-HCH-induced chemoresistance.

### 2.4. β-HCH Counteracts TKIs’ Effects on Colony Formation

**Figure 7:**
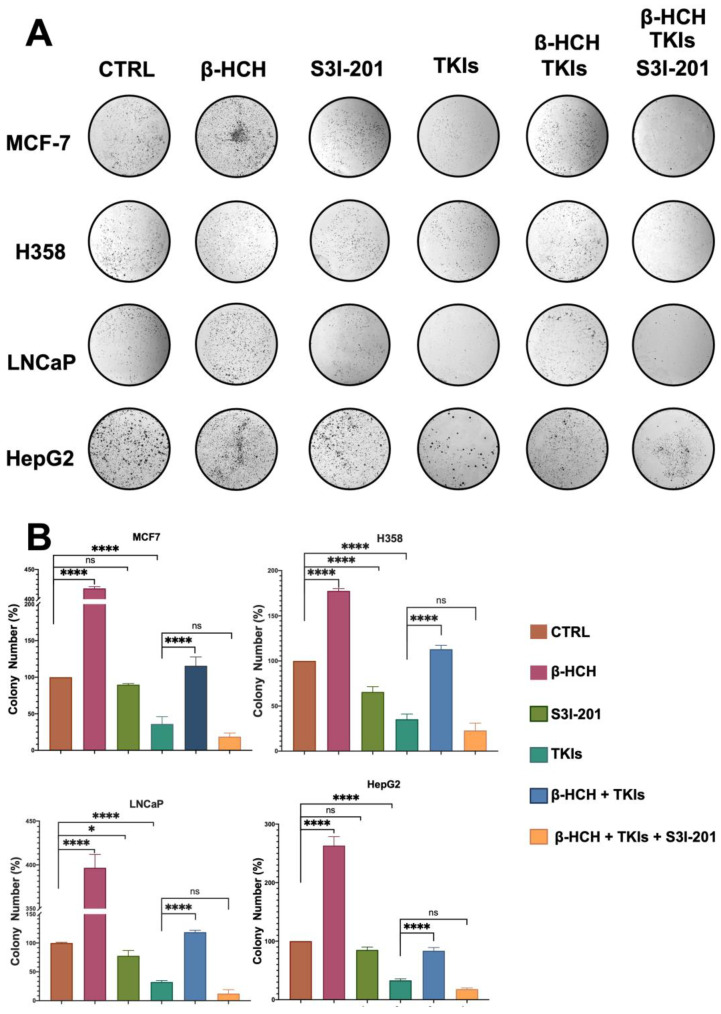
Clonogenic assay conducted on MCF-7, H358, LNCaP, and HepG2 cell lines. β-HCH induced an increase in colony formation, and in the triple treatment (β-HCH+ TKI+ S3I-201), by inhibiting the STAT3 protein, there was a reduction in cellular colony formation. The cells were pretreated with 10 µM of β-HCH in flasks for 7 days and then seeded at a density of 500 cells/mL in 6-well plates and cotreated for 5 days with specific TKIs, as shown in Figure 2. After treatments, the colonies formed were evidenced using crystal violet dye (Panel **A**) and counted, and the total areas of colonies (expressed as percentages with respect to the control and SD) are shown in the histogram (Panel **B**). These images are representative of three independent experiments with similar results. Statistical analysis was performed with the GraphPad Prisma software using ANOVA followed by Tukey’s post hoc test. Statistically significant differences were determined at * *p* < 0.05; and **** *p* < 0.0001, ns: not statistically significant.

A clonogenic cell survival assay determines the ability of a cell to proliferate indefinitely, thereby retaining its reproductive ability to form a large colony or a clone [44]. Due to its characteristics, this assay is useful for studying the inhibitory action of a drug or the carcinogenic effect of a compound. In our study, this assay allowed us to confirm what has already been observed via viability and migration assays. As shown in Figure 7, the colony formation capability of the four cell lines followed a similar trend to that observed in the migration assay. In the presence of β-HCH, the number of colonies increased compared with the control. Treatment with the drugs reduced the clonogenicity, while the efficacy of the drugs was reduced in the co-treatment (β-HCH + TKIs). Conversely, in the triple treatment where the STAT3 protein was inhibited, the efficacy of the drugs was restored.

## 3. Discussion

Chemoresistance in cancer therapies represents the major therapeutic failure responsible for disease progression and eventually death. Ongoing research is therefore increasingly engaged in overcoming this clinically relevant issue by developing pharmacological agents targeting new molecular targets and/or devising combinations with agents capable of inhibiting the onset of chemoresistance [45]. The response of a human organism to a specific therapy is often estimated without considering the drug’s interaction with other xenobiotic compounds, i.e., environmental pollution. As supported by well-established scientific evidence, chemical carcinogenesis may contribute to, or even be responsible for, the phenomenon of chemoresistance [46]. Examining the mechanisms that induce acquired resistance, it was hypothesized that compounds capable of activating cellular responses such as proliferation and the activation of oncogenic and anti-apoptotic pathways may contribute to predisposing cancer cells to non-sensitivity to chemotherapy. β-HCH is a pollutant belonging to the OCPs family, and the cellular mechanisms underlying its action correspond to many of those inducing chemoresistance. It has been verified via cellular studies that β-HCH counteracts the effectiveness of chemotherapeutic agents. For this study, four cell lines representative of the most frequent tumors were used. β-HCH’s experimental concentration was extrapolated from an epidemiological study [31] and the concentrations of the specific inhibitors from the scientific literature [47,48,49,50]. All obtained results clearly showed a reduced cellular response to the chemotherapeutic agent when the cell samples were exposed to β-HCH. The viability assay demonstrated a decrease in cell death in samples subjected to co-treatment with the drug and β-HCH, and this was confirmed by the migration and clonogenic assays.

The activation of oncogenic pathways investigated via Western blotting supports the cellular responses. In samples pretreated with β-HCH and then with the TKIs, there was an evident activation of oncogenic pathways, in particular mediated by the STAT3 protein, as we have already shown in previous works [20].

In our opinion, the results presented in this study are relevant because we utilized the concentration of β-HCH detected in the plasma of inhabitants undergoing an epidemiological surveillance study in Valle del Sacco.

It is evident that at this concentration, there is a reduction in the efficacy of TKIs in all cellular lines representing four different tumors. In contaminated areas, β-HCH is not the only pollutant. There are several contaminants such as particulate matter 2.5 (PM2.5), asbestos, heavy metals, etc., and the synergistic activity of these pollutants increases cell damage, with serious implications for human health, as described in the literature [51]. These results point toward a different therapeutic approach for cancer patients living in polluted territories, which will include the identification and measurement of environmental pollutants’ plasmatic levels in these patients. In this way, a more personalized treatment could be provided, predicting the best therapies based on the type of cancer within the context of the environmental pollutants to which the patient is exposed.

## 4. Materials and Methods

### 4.1. Cell Cultures

**Figure 8:**
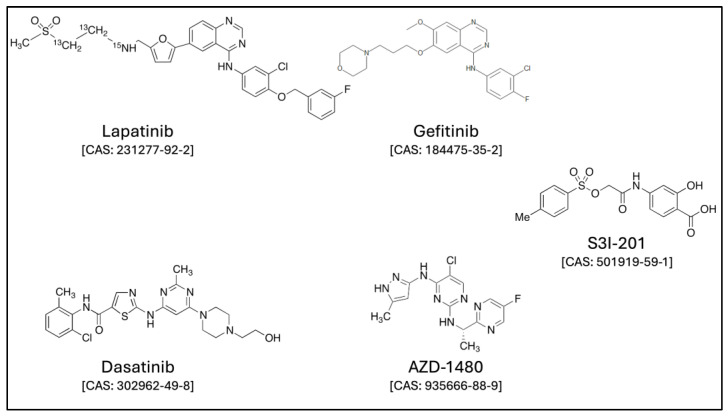
The available structures were obtained from the following link: https://www.lgcstandards.com/ (accessed on 29 May 2024).

The human breast cancer cell line MCF-7, human bronchoalveolar cancer cell line H358, human prostate cancer cell line LNCaP, and human hepatoma cell line HepG2 were obtained from the American Type Culture Collection (ATCC, Manassas, VA, USA). The cells were cultured until approximately 70% to 90% confluent, in a 95% air/5% CO_2_ humidified atmosphere using a proper culture medium, RPMI 1640 (Sigma-Aldrich, Milan, Italy, Cat. No. R0883) or DMEM-LG (Sigma-Aldrich, Milan, Italy, Cat. No. D5546), supplemented with 1% sodium pyruvate, 10% fetal bovine serum, 2 mM glutamine, 100 μg/mL streptomycin, and 100 U/mL penicillin. All four cell lines were treated with β-hexachlorocyclohexane (β-HCH) (Sigma-Aldrich, Milan Italy, Cat. No. 33376), with a final concentration of 10 μM. For the experiments, the following inhibitors (Figure 8) were used at the specified concentrations: 0.8 μM lapatinib (Sigma-Aldrich, Milan, Italy, Cat. No. CDS022971), 15 µM gefitinib (Sigma-Aldrich, SLM1657), 70 nM dasatinib (Selleckchem, Roma, Italy, Cat. No. S1021), 6 μM AZD1480 (Sigma-Aldrich, Milan, Italy, Cat. No. SML1505), and 100 μM S3I-201 (Sigma-Aldrich, Milan, Italy, Cat. No. SML0330).

### 4.2. Cell Viability

The impact of 10 μM of β-HCH for 12 h on cell viability was evaluated by seeding 12,000 cells/well in 96-well plates. According to the manufacturer instructions, after the necessary incubation time with β-HCH and/or anticancer drugs, cell viability was measured using the Cell Counting Kit-8 (CCK-8) (MedChemExpress, Guidonia Montecelio, Italy, Cat. No. HY-K0301). Briefly, the culture medium was removed, and 125 μL of CCK-8 solution was added to each well. After 3 h of incubation, the plate was gently mixed with an orbital shaker for 1 min to ensure a homogeneous color distribution, and the absorbance was measured at 570 nm using the Appliskan plate reader (Thermo Fisher Scientific, Monza, Italy).

### 4.3. Protein Extraction and Immunoblotting

Protein extraction and Western blotting analysis were performed according to Cocchiola et al. [52]. In summary, cells at a density of 300,000 cells/well were scraped, centrifugated, and washed in PBS (Sigma-Aldrich, Milan, Italy, cat. D8662). Total cellular protein was extracted using a buffer solution composed of 2% SDS, 20 mM Tris-hydrochloride at pH 7.4, 2 M urea, 10% glycerol supplemented with 2 mM sodium orthovanadate, 10 mM DTT, and a protease inhibitor cocktail diluted to 1:100 (Sigma-Aldrich, Milan, Italy). The cellular proteins were analyzed by SDS-PAGE 10% TGX FastCastTM acrylamide gel (BioRad, Segrate, Italy, cat. No. 161-0183) and transferred to PVDF membranes using the Trans-Blot^®^ Turbo^TM^ Transfer System (BioRad, Segrate, Italy, cat. No. 170-4247). The blotting membranes were incubated overnight with 3% *w*/*v* non-fat dried milk or 0.2% *w*/*v* I-block (Thermo Fisher Scientific, T2015, Monza, Italy) in Tris-buffered saline containing 0.05% Tween-20 (TBS-T) and then with a specific primary antibody for 1 h. In the next step, the membranes were washed three times in I-block and treated for one hour with specific anti-antibodies that had been conjugated to alkaline phosphatase (Sigma-Aldrich, Milan, Italy, Cat. No. A3687-A3688, dilution 1:5000). The alkaline phosphatase was detected with tetrazolium salt and the indoxyl phosphate (BCIP/NBT reagents) (Carl Roth, Milan, Italy, Cat. No. 6368.1 and 4421.3). The densitometric analysis was performed by the ImageLab Software, version 6.0.1 after the acquisition of the respective images with the Molecular Imager^®^ ChemiDoc™ MP System (Bio-Rad, Segrate, Italy). The antibodies used in the Western blotting analysis are listed as follows: anti-STAT3 (Cell Signaling, Pero, Italy, Cat. No. 9132), anti-pY705-STAT3 (Cell Signaling, Pero, Italy, Cat. No. 9145), anti-p-SRC (Cell Signaling, Pero, Italy, Cat. No. 6943), anti-Src (Cell Signaling, Pero, Italy, Cat. No. 2108), anti-β-actin (Sigma-Aldrich, Milan, Italy, Cat. No. A1978 clone AC-15), anti-pY1007/1008-Jack2 (Cell Signaling, Pero, Italy, Cat. No. 3776), anti-Jack2 (Cell Signaling, Pero, Italy, Cat. No. 3230), anti-pHER2 (Cell Signaling, Pero, Italy, Cat. No. 2247S), and anti-HER2 (Cell Signaling, Pero, Italy, Cat. No. 2242). All primary antibodies were diluted according to the manufacturer’s instructions. Each experiment was replicated at least three times. β-actin (total extracts) was used as a normalization protein.

### 4.4. Wound-Healing Assay

In order to assess the proliferative activity, a wound-healing assay was performed [53]. The MCF-7, H358, LNCaP, and HepG2 cell lines were seeded in 6-well plates at a density of 300,000 cells/well. After adherence, the cells were pretreated with β-HCH for 12 h. Following the 12-h treatment, a scratch was made at the bottom of each well using a pipette tip. At this point, defined as T0, images were captured, and the TKIs were added. After 8 h, the cells were also treated with S3I-201. The final images were captured after 48 h of treatment with the TKIs with a Leica AF6000 Modular System microscope (Leica Microsystem, Milan, Italy).

### 4.5. Colony Formation Assay

The cells were seeded at a density of 200 cells/mL in 6-well plates and treated for one week with 10 μM of β-HCH and for a further two weeks in the presence or absence of specific TKIs and S3I-201. The medium was removed, and then the cells were rinsed with PBS and fixed with cold MeOH for 30 min at 4 °C. Thereafter, the colonies were stained by incubating the cells with a mixture of 1% crystal violet in 25% MeOH for 1 h at room temperature. After the removal of the staining solution, each well was washed with abundant H_2_O and air-dried at room temperature. The colonies were counted using the ImageJ software according to Rubini et al. [54].

### 4.6. Statistical Analysis

All of the data are expressed as the means ± standard error (SE) of at least three biological replicates, in which at least two technical replicates per concentration were performed. The statistical analysis was carried out by the GraphPad Prism™ software (Version 8.2.1(279), GraphPad Software, Inc., San Diego, CA, USA). One-way ANOVA, followed by Tukey’s post hoc test, was used to analyze the differences between treatments. A *p*-value of <0.05 was considered significant.

## 5. Conclusions

This cellular study showed that β-HCH, one of the global persistent environmental pollutants belonging to the OCPs family, by contributing to the induction of chemoresistance, may reduce the efficacy of TKIs. These results strongly support the need to extend this type of investigation to other OCPs. Furthermore, they may have relevant translation implications since they indicate that the selection of chemotherapeutic regimens in cancer patients with documented exposure in contaminated areas should eventually undergo rescheduled tailored treatment. The present data, due to the scarcity of reports in the scientific literature regarding prolonged exposure to contaminants and their impact on drug efficacy, underline that this issue requires increasing attention at the scientific, healthcare, and political levels.

## Data Availability

The data are contained within this article.
